# 20(*S*)‐Protopanaxadiol inhibits epithelial‐mesenchymal transition by promoting retinoid X receptor alpha in human colorectal carcinoma cells

**DOI:** 10.1111/jcmm.16054

**Published:** 2020-10-30

**Authors:** Zeyuan Lu, Hongyan Liu, Wenwen Fu, Yuchen Wang, Jianan Geng, Yaozhen Wang, Xiaofeng Yu, Quan Wang, Huali Xu, Dayun Sui

**Affiliations:** ^1^ Department of Pharmacology School of Pharmaceutical Sciences Jilin University Changchun China; ^2^ Department of Gastrocolorectal Surgery First Affiliated Hospital of Jilin University Changchun China

**Keywords:** 20(*S*)‐protopanaxadiol, colorectal carcinoma, epithelial‐mesenchymal transition, metastasis, retinol X receptor alpha

## Abstract

Colorectal carcinoma (CRC) recurrence is often accompanied by metastasis. Most metastasis undergo through epithelial‐mesenchymal transition (EMT). Studies showed that retinol X receptor alpha (RXRα) and 20(*S*)‐Protopanaxadiol (PPD) have anti‐tumour effects. However, the anti‐metastasis effect of 20(*S*)‐PPD and the effect of RXRα on EMT‐induced metastasis are few studies on. Therefore, the role of RXRα and 20(*S*)‐PPD in CRC cell metastasis remains to be fully elucidated. RXRα with clinicopathological characteristics and EMT‐related expression in clinical samples were examined. Then, RXRα and EMT level in SW480 and SW620 cells, overexpressed and silenced RXRα in SW620 cells and SW480 cells, respectively, were evaluated. Finally, 20(*S*)‐PPD effect on SW620 and SW480 cells was evaluated. The results showed that a lower RXRα expression in cancer tissues, and a moderate negative correlation between RXRα and N stage, and tended to higher level of EMT. SW480 and SW620 cells had the highest and lowest RXRα expression among four CRC cell lines. SW480 had lower EMT level than SW620. Furthermore, 20(*S*)‐PPD increased RXRα and inhibited EMT level in SW620 cell. Finally, 20(*S*)‐PPD cannot restore SW480 cells EMT level to normal when RXRα silencing. These findings suggest that 20(*S*)‐PPD may inhibit EMT process in CRC cells by regulating RXRα expression.

## 1 INTRODUCTION

Colorectal carcinoma (CRC) ranks the third in incidence but the second in mortality. In 2020, approximately 147 950 individuals will be diagnosed with CRC and 53 200 will die from the disease.^1^ When patients are diagnosed at the early stages of CRC, the 5‐year survival rate is 90%.^2^ Most patients with primary tumours undergo surgical resection combined with chemotherapy and radiotherapy as the preferred treatment. CRC patients often experience constipation, diarrhoea or rectal bleeding. Some patients may exhibit symptoms such as weakness and fatigue, but many patients do not exhibit symptoms in early stages. In addition, there is lack of awareness regarding screening of CRC in most regions of China. The above characteristics of colorectal cancer and the limitations of public perception of colorectal cancer contribute to late‐stage diagnosis when the tumour has already metastasized. Most patients reach the advanced stage and exhibit metastasis.^3^ Screening of metastatic markers and targeted therapy for metastatic colorectal cancer are essential.

Epithelial‐mesenchymal transition (EMT) is a process in which an epithelial cell converts into a mesenchymal cell and obtains the ability of cell invasion and motility. Studies showed that EMT plays an important role in invasion and metastasis of malignant cancer.^4^ During EMT, the invasiveness properties of mesenchymal cells are characterized by the down‐regulating epithelial markers (E‐cadherin) while up‐regulating mesenchymal markers (N‐cadherin, vimentin) expression. Additionally, transcription factors like Snail, Slug and ZEB‐1 are up‐regulated for promoting EMT process or inhibiting epithelial marker transcription.^4^

Retinoid X receptor (RXR)is a type of nuclear receptor (NR). There are three isoforms of RXR: α, β and γ. Recently, some studies showed irregular expression of RXRα in various cancer tissues. The expression of RXRα mRNA was lower in oesophageal cancer tissue than that in Barrett's oesophageal tissue in patients with extensive lymph node metastasis.^5^ A clinical study showed that RXRα agonist is a potential drug for cancer prevention and malignancies.^6^ At present, the correlation between the expression of RXRα and tumour metastasis caused by EMT was not clearly studied.

20(*S*)‐Protopanaxadiol (PPD) is an active ginseng metabolite (Figure S1), which is the final form of protopanaxadiol saponins metabolized by human intestinal microflora.^7^ It was reported that 20(S)‐Protopanaxadiol saponins (with ginsenosides Rb1, Rb3 and Rd) inhibit migration in SKOV3 cell.^8^ Moreover, some ginsenosides showed their functions like inhibiting angiogenesis, prostate cancer prevention by regulating NRs.^9^, ^10^ As a metabolite of protopanaxadiol saponins, 20(*S*)‐PPD also performed a role in a variety of cancer inhibition and NR regulation.^11^, ^12^ It showed that 20(*S*)‐PPD can promote the concentration of calcitriol,^13^ inhibiting castration‐resistant prostate cancer by regionally targeting androgens.^14^ According to current studies, 20(*S*)‐PPD anti‐tumour effect is caused by its cytotoxicity which leads to the apoptosis of various cancer cells.^15^, ^16^ However, the anti‐metastatic effect of 20(*S*)‐PPD and the effect of 20(*S*)‐PPD on RXRα and EMT level in colorectal cancer cells are largely unknown.

Based on the above researches, we speculated that 20(*S*)‐PPD could regulate one or more NRs and its (theirs) downstream pathway(s). Meanwhile, based on the anti‐tumour effect of 20(*S*)‐PPD, it was speculated that it could inhibit the EMT process of tumour cells. And the mechanism of inhibiting EMT may be related to NR pathway, which was regulated by 20(*S*)‐PPD. Thus, in this study, the relationship between RXRα expression and EMT level was examined, and whether RXRα expression regulated tumour metastasis. Also, 20(*S*)‐PPD anti‐metastasis effect was investigated.

## 2 MATERIALS AND METHODS

### 2.1 Cell lines and reagents

HCT‐116, SW480, SW620 and LoVo human colorectal cancer cell lines were purchased from MeiXuan Biotech. All four cell lines were cultured in high glucose Dulbecco's modified eagle medium (DMEM) with 10% foetal bovine serum (FBS) (both from Gibco; Thermo Fisher Scientific). These cells were incubated at 37°C and 5% CO_2_. Radio immunoprecipitation assay (RIPA) lysis buffer and phenylmethylsulfonyl fluoride (PMSF) and 4% paraformaldehyde were purchased from Dingguo Changsheng Biotechnology Co. Ltd. BCA protein assay reagent kit and ECL chemiluminescence reagent were purchased from Beyotime Institute of Biotechnology. TRIzol and Lipofectamine 3000 were bought from Invitrogen (Thermo Fisher Scientific). RXRα antibody and Epithelial‐Mesenchymal Transition Antibody Sampler Kit (including E‐cadherin, N‐cadherin, vimentin, β‐catenin, Snail, Slug and horseradish peroxidase [HRP]‐linked Anti‐Rabbit IgG antibody) were bought from Cell Signaling Technology Inc (USA). RXRα, E‐cadherin, vimentin, Snail and ZEB‐1 antibodies for immunohistochemistry (IHC) were purchased from Abcam. GAPDH was purchased from Zhongshan Jinqiao Biotechnology Co., Ltd. Anti‐Rabbit, Anti‐Mouse and Anti‐Goat IgG HRP‐linked secondary antibodies were bought from Dingguo Changsheng Biotechnology Co., Ltd. pBABE‐puro human RXRα plasmid was benefited from Ronald Kahn Lab from the Addgene website. Plasmid extraction kit were bought from TransGen Biotech Co., Ltd. RXRα, E‐cadherin, Snail, ZEB‐1 and GAPDH RNA primers were purchased from Dingguo Changsheng Biotechnology Co., Ltd. The RXRα siRNA sequence was bought from Sangon Biotech Co., Ltd. 20(*S*)‐PPD was provided by Hainan Asia Pharmaceutical Co. Ltd. The purity of 20(*S*)‐PPD used in experiments was 99.67%, as determined by high‐performance liquid chromatography.

### 2.2 Spleen subcapsular injection colorectal cancer liver metastasis models

Nude mice were provided by Beijing Vital River Laboratory Animal Technology Co., Ltd. The method of spleen subcapsular injection colorectal cancer liver metastasis models was based on the method described previously.^17^ Briefly, a longitudinal incision was made under the left costal margin and then the spleen was exposed. SW480 and SW620 cell suspensions were adjusted to 1 × 10^7^/mL with PBS, and 0.2 mL cell suspension was injected between the subcapsular and parenchyma of the spleen, respectively. Before injection, cells viabilities were measured by trypan blue staining based on this protocol to ensure at an equivalent viability levels of these cells.^18^

Nude mice were administered 20(*S*)‐PPD intragastrically daily for 4 weeks. According to our previous study, the dosages of 20(*S*)‐PPD were 50 mg/kg and 100 mg/kg in the low‐dose and high‐dose groups, respectively.^19^ The mice were killed 1 hour after the last administration. The metastatic nodules on the liver of nude mice were counted and photographed, and then livers were fixed with 10% formalin.

Animals were treated according to the Guide for the Care and Use of Laboratory Animals [United States National Institutes of Health (NIH)] and the Committee for the Care and Use of Laboratory Animals of Jilin University (Changchun, China). The study protocol was approved by the Ethics Committee of Jilin University (20190045).

### 2.3 Patients and specimens

A total of 20 patients who were diagnosed with colorectal carcinoma were investigated. Written informed consent was obtained from all patients. All protocols in this part of investigation were approved by the first affiliated hospital of the Jilin University Ethics Committee according. The research was conducted in accordance with the 1964 Declaration of Helsinki and its later amendments. No patient received any neoadjuvant chemotherapy or radiotherapy before colorectum resection (2017ZSLYEC‐015). Specimens of both carcinoma and para‐carcinoma tissues that were obtained during colorectum resection were immediately stored in a 10% neutral formalin solution for further analysis by IHC.

### 2.4 Cell transfection

SW620 and SW480 cells were seeded in 6‐well plates with 3 × 10^5^ cells/mL and incubated at 37°C and 5% CO_2_ for 24 hours. RXRα overexpression plasmid or RXRα siRNA (Sense: 5′‐GCG CCA UCG UCC UCU UUA ATT‐3′, Antisense: 5′‐UUA AAG AGG ACG AUG GCG CTT‐3′) were mixed with Opti‐MEM (Gibco; Thermo Fisher Scientific) and transfected by Lipofectamine‐3000 in SW620 cell or SW480 cell, respectively, and the transfected cells were incubated at 37°C and 5% CO_2_ for 24 hours.

### 2.5 Cell wound scratch assay

Cell concentration was adjusted to 1 × 10^6^ cells/mL and plated into 6‐well plates. The cells were grown till confluence, and a straight line was scratched in each well with 10 μL tips; subsequently, the cells were incubated in serum‐free medium for 24 and 48 hours. The healing of the wound was observed under the Nikon TE‐2000U optical microscope (Nikon Corporation).

### 2.6 Cell migration and invasion assay

Cells were plated at a concentration of 2 × 10^5^ cells/ml in a 24‐well Transwell plate (Greiner Bio‐One) with 8.0 μm pore, and 100 μL of the serum‐free medium was added. In the lower chamber, 500 μL of medium including 10% FBS was added. The plates were incubated at 37°C and 5% CO_2_ for 12‐18 hours. Cells on the upper side of the membrane were wiped off, and cells on the lower side of the membrane were fixed with 4% paraformaldehyde and stained with crystal violet. The number of cells on the lower side were counted under the Nikon TE‐2000U inverted microscope. ImageJ (version 1.5.0.26; National Institutes of Health) was used for analysis.

### 2.7 Western blot

Protein extraction and Western blot were performed as described previously.^7^ Briefly, Western blot was performed for the detection of RXRα (cat. no. 3085S), E‐cadherin, N‐cadherin, β‐catenin, vimentin, Snail, Slug, claudin‐1 and ZEB‐1 (all from cat. no. 9782T), and GAPDH (cat. no. TA‐08). Cells were collected and lysed. Protein concentration was determined using the BCA protein assay kit. And proteins were loaded on polyacrylamide‐SDS gel (30 µg/lane), blotted onto a PVDF membrane and blocked with non‐fat milk for 1 hour at room temperature. The membrane was incubated overnight with primary antibody at 4°C, followed by incubation with HRP‐conjugated secondary antibody: goat‐anti‐mouse (cat. no. IH‐0031) and goat‐anti‐rabbit (cat. no. IH‐0011) at room temperature for 1 hour. The blots were visualized by BeyoECL Plus enhanced chemiluminescence kit (Beyotime Institute of Biotechnology; Jiangsu, China). ImageJ software was used for analysis.

### 2.8 IHC assay

The slices of colorectal cancer tissues and para‐carcinoma tissues from patients were deparaffinized with xylene and rehydrated with ethanol. Then, the antigen was unmasked with citrate buffer. Subsequently, the slices were blocked and incubated overnight in primary antibodies against RXRα (cat. no. ab191176), E‐cadherin (cat. no. ab76055), Snail (cat. no. ab53519), vimentin (cat. no. ab92547) and ZEB‐1 (cat. no. ab180905) 4°C, followed by incubation in secondary antibodies from two‐step kits (PV‐6001, PV‐9003; Zhongshan Jinqiao Biotechnology co. LTD). DAB (ZLI‐9017; Zhongshan Jinqiao Biotechnology co. LTD) was used for immunostaining, and nuclei were stained with haematoxylin (AR‐0712; Dingguo Changsheng Biotechnology Co. Ltd). Slices were observed under the Nikon Eclipse 80i fluorescence microscope. ImageJ software was used for analysis.

### 2.9 Quantitative reverse‐transcription polymerase chain reaction (qRT‐PCR)

The extraction, isolation and purification of RNAs were performed with the TRIzol reagent. TransScript Green Two‐Step qRT‐PCR SuperMix (AQ201; TransGen Biotech Co., LTD) was used for cDNA synthesis and amplification. GAPDH was used as an internal control in each group to normalize the variability at mRNA expression levels. All samples were tested by MX3000p and MXPro 4.0 (Stratagene, Agilent). The sequence of the primers used were as follows: GAPDH (F: 5′‐AGAAGGCTGGGGCTCATTTG‐3′, R: 5′‐AGGGGCCATCCACAGTCTTC‐3′), E‐cadherin (F: 5′‐ATGCTGATGCCCCCAATACC‐3′, R: 5′‐GGGGGCTTCATTCACATCCA‐3′), RXRα (F: 5′‐ACACCAAACATTTCCTGCCG‐3′, R: 5′‐TGTTGGTGACAGGGTCGTTC‐3′), Snail (F: 5′‐TAGCGAGTGGTTCTTCTGCG‐3′, R: 5′‐AGATGAGCATTGGCAGCGAG‐3′), vimentin (F: 5′‐GAGAACTTTGCCGTTGAAGC‐3′, R: 5′‐GCTTCCTGTAGGTGGCAATC‐3′), β‐catenin (F: 5′‐ACGGAGGAAGGTCTGAGGAG‐3′, R: 5′‐CCAAGCATTTTCACCAGGGC‐3′), claudin‐1 (F: 5′‐AGCGGGAAAGACTACGTGTG‐3′, R: 5′‐GGGATTGTGTGGGAAGGTCA‐3′) and ZEB‐1 (F: 5′‐AGAGCGCTAGCTGCCAATAA‐3′, R: 5′‐GGGCGGTGTAGAATCAGAGT‐3′). The quantity of E‐cadherin, RXRα, Snail, vimentin, β‐catenin, claudin‐1 and ZEB‐1 were calculated using the equation [RQ = 2^−ΔΔCT^, ΔΔCT = [(CT _group 1_) − (CT_SW620 or SW480 group_)] − [(CT _group 2_) − (CT_SW620 or SW480 group_)]], Six replicates were taken for each group.

### 2.10 MTT assay

Briefly, SW480 and SW620 were seeded in separate 96‐well plates and incubated at 37°C and 5% CO_2_ for 24 hours. Subsequently, the cells were treated with different concentrations of 20(S)‐PPD for another 20 hours, and the MTT reagent was added in each well and incubated for 4 hours. Finally, the crystals were dissolved in DMSO. The OD value from each well was read, and the cell viability was determined.

### 2.11 Statistical analysis

The results were expressed as mean ± standard deviation (SD). Statistical differences were evaluated using a two‐tailed Student *t* test and one‐way analysis of variance (ANOVA) with the SNK post hoc test. *P* < .05 was considered statistically significant. Correlation analysis was performed with Pearson's method. SPSS 22 statistical software (IBM Corp) was used for analysis.

## 3 RESULTS

### 3.1 Correlation of RXRα expression with clinicopathological characteristics in colorectal cancer patients

**Figure 1:**
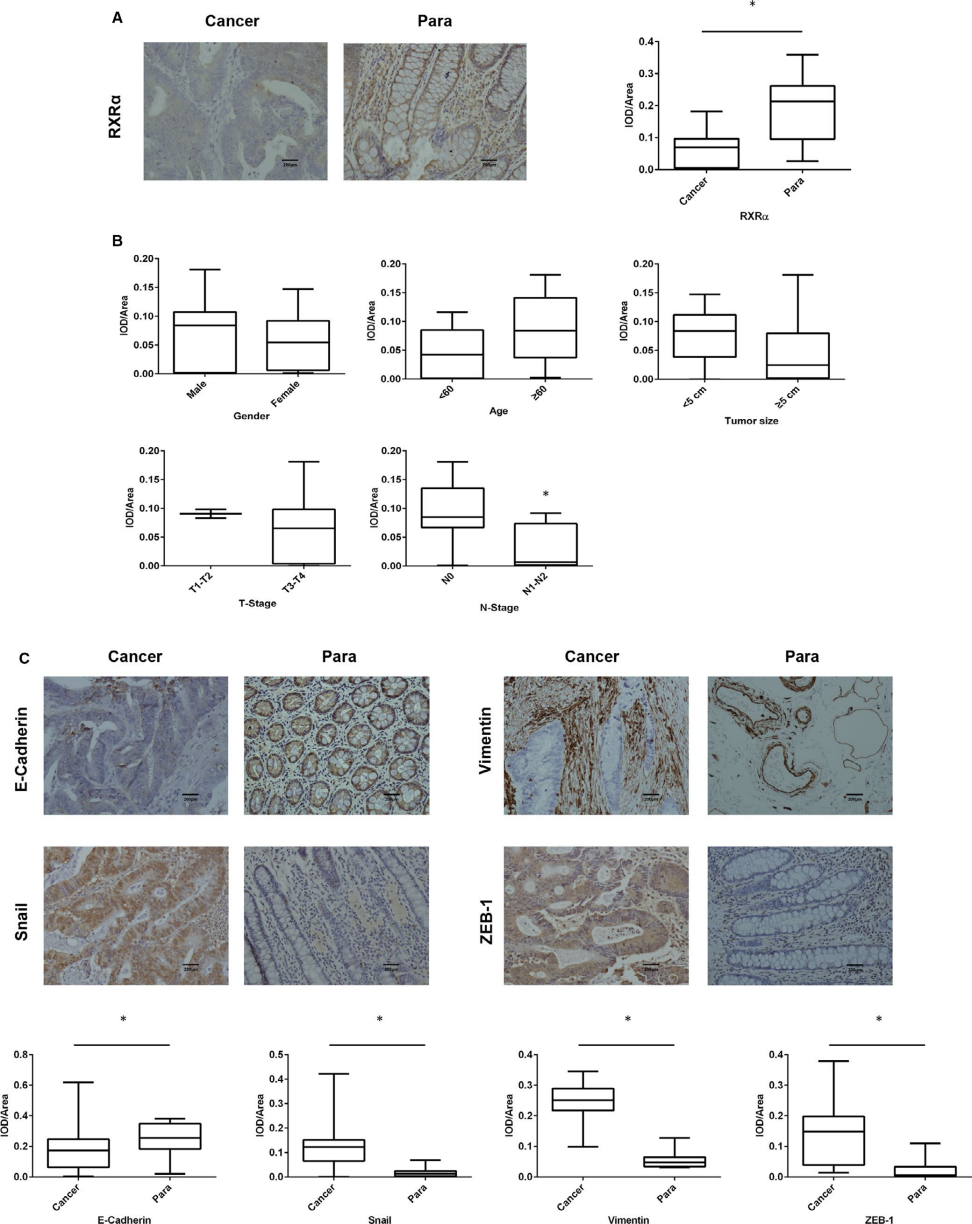
RXRα‐ and EMT‐related proteins expression in cancer and para‐carcinoma tissues. A, RXRα immunohistochemical staining and positive staining in cancer tissues and para‐carcinoma tissues of CRC patients. Three randomized fields at a magnification of 200×. *P < .05 presented a significant difference between cancer tissues and para‐carcinoma tissues. Cancer, cancer tissues; Para, para‐carcinoma tissues. B, RXRα‐positive expression area in cancer tissues of CRC patients according to gender, age, tumour size, T stage, N stage, M stage and their respective subgroups. C, E‐cadherin, vimentin, Snail and ZEB‐1 immunohistochemical staining and positive staining in cancer and para‐carcinoma tissues of CRC patients. Three randomized fields at a magnification of 200×. The data represented as mean ± SD. All results were statistically analysed using a two‐tailed Student *t* test. *P < .05 represented significance. Cancer, cancer tissues; Para, para‐carcinoma tissues

As a result of the abnormal expression of RXRα in various cancers, the differences in RXRα expression were determined between 20 pairs of colorectal cancer tissue and para‐carcinoma tissue. The RXRα expression was significantly lower in cancer tissues than in para‐carcinoma tissues (Figure 1A). Moreover, the difference in RXRα expression in these two types of tissues according to the clinicopathological characteristics was analysed. A significant difference in the RXRα expression between cancer and para‐carcinoma tissue was observed in all subgroups including gender, age, tumour size and TNM stage except the T0 stage and M1 stage (Table 1). In addition, we found that in cancer tissues, the expression of RXRα was significantly different between N0 and N1‐N2 stages (Figure 1B and Table 1). There was a moderate negative correlation only between RXRα expression and N stage which represents the level of lymph node metastasis (Table 2). We examined the expression of EMT‐associated markers and found that the E‐cadherin expression was significantly lower in cancer tissues than in para‐cancer tissues while the expression of vimentin, Snail and ZEB‐1 was notably increased in cancer tissues (Figure 1C). These findings indicated that RXRα was decreased in cancer tissue and a decrease in RXRα might induce EMT development and promote lymph node metastasis.

**Table 1:** The RXRα expression in tumour tissues, para‐tissues and clinicopathological features

Characteristics	Case(s)	Tumour RXRα expression	*P* value	Para‐tissue RXRα expression	*P* value
Gender			.496		
Male	10	0.0740 ± 0.0600		0.1672 ± 0.1093	.000*
Female	10	0.0569 ± 0.0495		0.2026 ± 0.0780	.002*
Age (y)			.091		
<60	11	0.0469 ± 0.0429		0.1962 ± 0.1021	.002*
≥60	9	0.0881 ± 0.0603		0.1711 ± 0.0873	.033*
Tumour size			.251		
<5 cm	12	0.0770 ± 0.0472		0.1595 ± 0.0932	.011*
≥5 cm	8	0.0480 ± 0.0625		0.2230 ± 0.0874	.003*
T stage			.506		
T1‐T2	2	0.0905 ± 0.0106		0.1915 ± 0.1209	.474
T3‐T4	18	0.0627 ± 0.0566		0.1842 ± 0.0951	.000*
N stage			.007^#^		
N0	11	0.0934 ± 0.0503		0.1712 ± 0.0820	.030*
N1‐N2	6	0.0313 ± 0.0380		0.2017 ± 0.1099	.001*
M stage			.126		
M0	19	0.0612 ± 0.0521		0.1862 ± 0.0966	.000*
M1	1	0.1470	—	0.1600	—

Note

The data represent the mean ± SD of at least three independent experiments. All resulting data were statistically analysed using a two‐tailed Student *t* test. *P < .05 was presented a significant difference between tumour RXRα expression and Para‐tissue RXRα expression under the sub‐features ^#^ *P *< .05 was presented a significant difference between tumour RXRα expression and each N stages.

**Table 2:** The correlation analysis between tumour RXRα expression and clinicopathological features

		Tumour RXRα expression	Tumour Size	T stage	N stage	M stage
Tumour RXRα expression	Pearson Correlation	1	−0.269	−0.158	−0.538	0.354
	*P* value		.251	.506	.014*	.126
Tumour Size	Pearson Correlation	−0.269	1	0.272	0.028	−0.187
	*P* value	.251		.246	.907	.429
T stage	Pearson Correlation	−0.158	0.272	1	0.272	0.076
	*P* value	.506	.246		.246	.749
N stage	Pearson Correlation	−0.538	0.028	0.272	1	−0.187
	*P* value	.014*	.907	.246		.429
M stage	Pearson Correlation	0.354	−0.187	0.076	−0.187	1
	*P* value	.126	.429	.749	.429	

Note

The data represent the correlation between tumour RXRα expression in all patients’ average and clinicopathological features. All resulting data were statistically analysed using Pearson correlation analysis. −0.6<‘Pearson correlation’ <−0.4 showed moderate negative correlation.

* *P *< .05 presented a significant difference.

### 3.2 Expression of RXRα in human CRC cells

**Figure 2:**
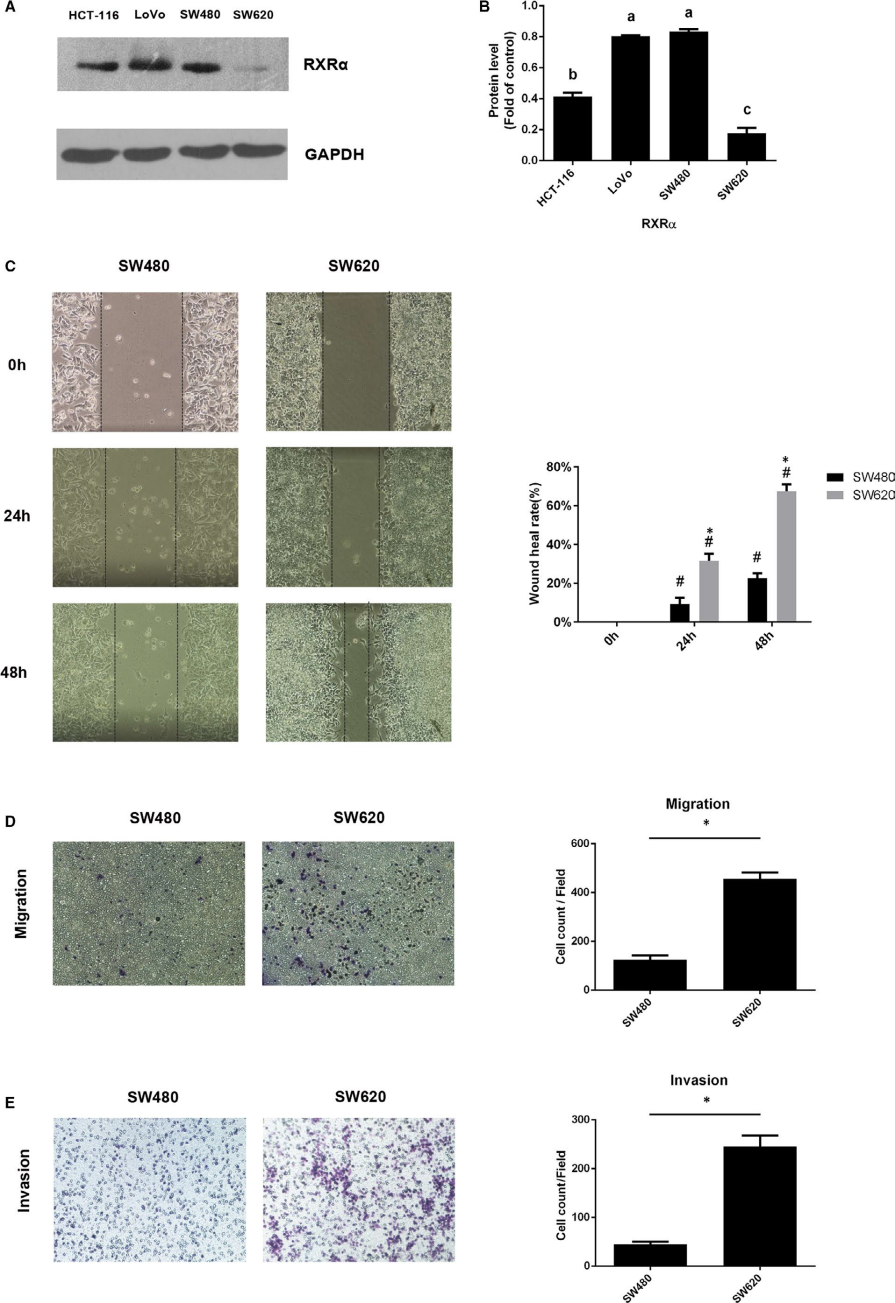
Difference in RXRα expressions and metastatic capabilities in several human CRC cells. A, B, Western blotting analysis of RXRα expression in HCT‐116, LoVo, SW480 and SW620 human CRC cells and quantification of Western blot analysis for RXRα expression. The data represents the means ± SD. ANOVA with SNK post hoc was used for analysis. There was a significant difference (*P* < .05) between groups identified by different letters. C, Cell wound scratch assay, (D) Transwell migration assay and (E) Matrigel invasion assays in SW480 and SW620 cells. The data represents as mean ± SD. C, D, all results were statistically analysed using a two‐tailed Student *t* test. *P < .05 represents a significant difference between SW480 and SW620 groups, and #*P *< .05 was in comparison with 0‐h time point in each group

Based on the previous result we found in 3.1, we assumed that the decrease in the expression of RXRα would lead to severe malignancy. HCT‐116, SW480, SW620 and LoVo cells with varying malignancy were harvested separately, and their proteins were extracted. The RXRα expression was examined using Western blot and was found to be different in four colorectal cancer cell lines (Figure 2A,B). RXRα protein level was the highest in SW480 cells and the lowest in SW620 cells. Thus, these two cell lines were used to explore the effect of RXRα on metastasis.

### 3.3 Migration and invasion capabilities in SW480 and SW620 cells

The cell wound scratch assay and transwell assay were used for evaluating cell metastasis capability. The wound healing capacity in 24 and 48 hours was higher in SW620 cells than in SW480 cells (Figure 2C). Consistently, the SW620 cells had higher invasive and stronger motility capability than SW480 cells, as more number of SW260 cells than SW480 cells crossed the membrane in the transwell assay (Figure 2D,E).

### 3.4 Expression of EMT‐associated proteins in SW480 and SW620 cells

**Figure 3:**
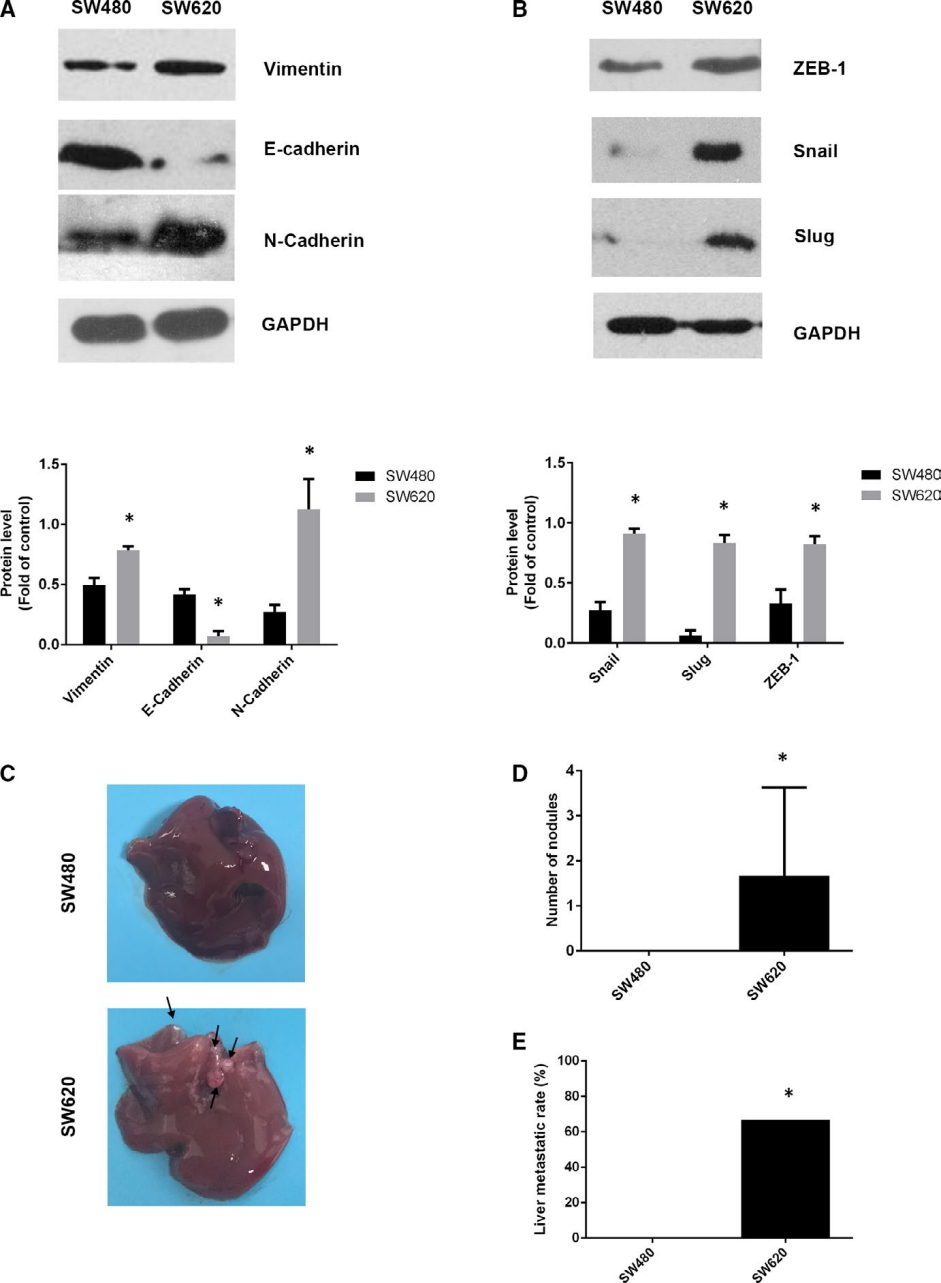
Epithelial‐mesenchymal transition‐associated proteins expressed in SW480 and SW620 cells. A, Vimentin, E‐cadherin, N‐cadherin and (B) ZEB‐1, Snail and Slug expressions in SW480 and SW620 cell lines were analysed by Western blot. C, Images of liver metastatic nodules (arrows indicate metastatic nodules) (D) Number of nodules and (E) liver metastatic rate in SW480 and SW620 injection nude mice model. The data represented as mean ± SD. All results were statistically analysed using a two‐tailed Student *t* test. *P < .05 represented a significant difference compared with the SW480 group

As EMT is one of the main pathways of tumour metastasis, it was necessary to analyse whether the expression of EMT marker proteins and transcription factors was altered in SW480 and SW620 cells. SW480 cells had significantly higher expression of E‐cadherin but significantly lower expression of N‐cadherin and vimentin than SW620 cells (Figure 3A). Additionally, the expression of transcription factors associated with EMT such as Snail, Slug and ZEB‐1 was lower in SW480 cells than in SW620 cells (Figure 3B). These results indicated that EMT was associated with strong migration and invasion capability, and SW620 had higher expression of EMT‐associated proteins than SW480 cells.

### 3.5 Capability of SW480 and SW620 cells for liver metastasis in vivo

To further assess the difference in metastatic capability between SW480 and SW620 cells, the number of liver metastatic nodules and metastasis occurrence rate was evaluated in the nude mice model as described previously. In nude mice livers, SW620 cells caused more severe metastasis than SW480 cells (Figure 3C). The occurrence rate of liver metastatic mice and the number of nodules were more in the case of SW620 cells than SW480 cells (Figure 3D,E). This result suggested that SW620 cells had a stronger metastatic capability than SW480 cells in vivo.

### 3.6 Overexpressed RXRα repressed EMT in SW620 cell

**Figure 4:**
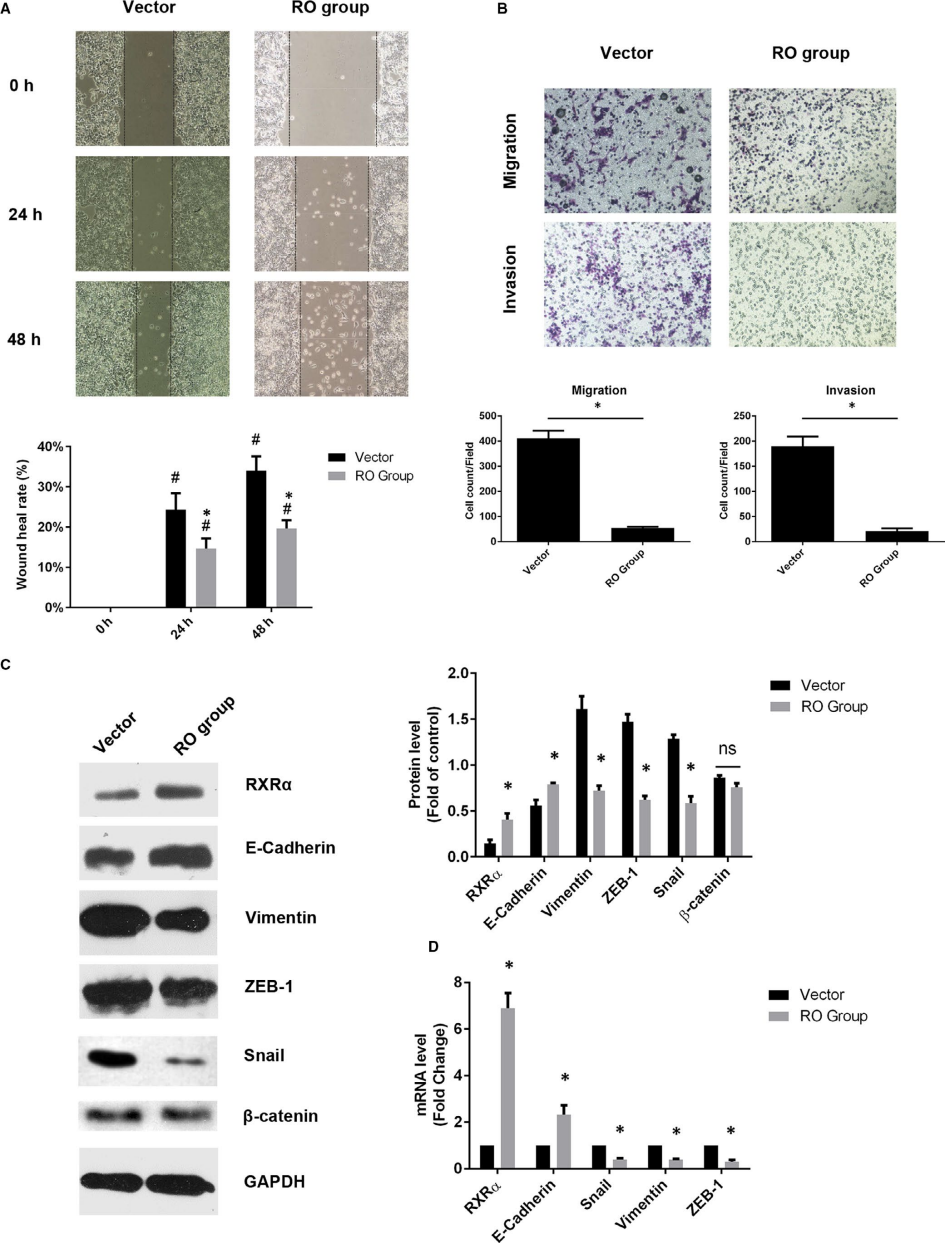
Overexpressed RXRα repressed epithelial‐mesenchymal transition in SW620 cells. A, Cell wound scratch assay and (B) Transwell migration and Matrigel invasion assays in RXRα overexpression SW620 cells. C, The protein level of RXRα, E‐cadherin, vimentin, Snail, β‐catenin and ZEB‐1 and (D) the mRNA level of RXRα, E‐cadherin, vimentin, Snail and ZEB‐1; The data represented as mean ± SD. All results were statistically analysed using a two‐tailed Student *t* test. *P < .05 represented a significant difference compared with the vector group, #*P *< .05 represented a significant difference compared with the 0‐h time point in each group. RO group: RXRα overexpressed SW620 group; ns, not significant

To demonstrate whether the expression of RXRα inhibited EMT in human CRC cells, we overexpressed RXRα in SW620 cells (RO group). The capability of migration and invasion were assessed by cell wound scratch and transwell assay. Overexpressed RXRα repressed the wound healing capability (Figure 4A) and cell mobility and invasion capability of SW620 cells (Figure 4B). These results suggested that increased RXRα would inhibit EMT in SW620 cells. Moreover, the expression of E‐cadherin was significantly higher but the expression of vimentin, Snail and ZEB‐1 was significantly lower in the RO group than in the vector group, yet β‐catenin expression had no difference between these two groups (Figure 4C). Consistently, qRT‐PCR results showed that expression of RXRα and E‐cadherin mRNA was up‐regulated whereas expression of Snail and ZEB‐1 mRNA was down‐regulated (Figure 4D). These results indicated that overexpression of RXRα repressed EMT in SW620 cells both at the protein and mRNA levels.

### 3.7 Deficient RXRα promoted EMT in SW480 cells

**Figure 5:**
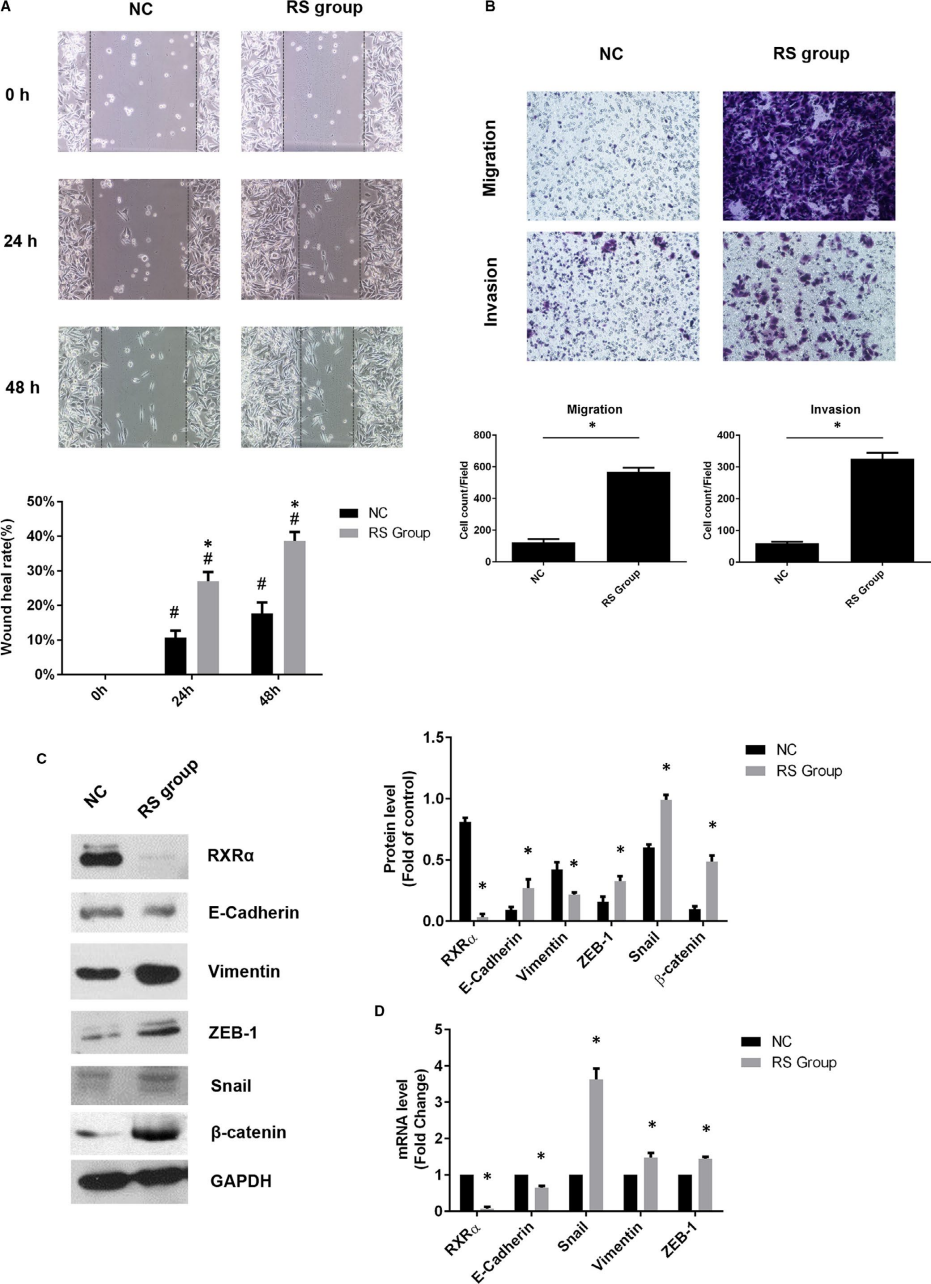
Deficient RXRα promoted epithelial‐mesenchymal transition in SW480 cells. A, Cell wound scratch assay and (B) Transwell migration and Matrigel invasion assays in RXRα silenced SW480 cells. C, The protein level of RXRα, E‐cadherin, vimentin, ZEB‐1, Snail and β‐catenin and (D) the mRNA level of RXRα, E‐cadherin, vimentin, Snail and ZEB‐1; The data represented as mean ± SD. All results were statistically analysed using a two‐tailed Student *t* test. *P < .05 represented a significant difference compared with the negative control group, #*P *< .05 represented a significant difference compared with the 0‐h time point in each group. NC, negative control; RS group, RXRα silenced SW480 group

To further determine the effect of RXRα deficiency on human CRC cells, we silenced RXRα expression with siRNA to establish RXRα silenced‐SW480 cells (RS group) and negative control cell (NC group). The expression of RXRα was significantly lower in the RS group than in the NC group (Figure 5C). The RS group had enhanced wound healing capability (Figure 5A). Moreover, the transwell assay showed that the RS group had higher cell mobility and invasion capability than the NC group (Figure 5B). Furthermore, the expression of E‐cadherin was down‐regulated while the expression of vimentin, Snail, β‐catenin and ZEB‐1 was up‐regulated in the RS group. (Figure 5C). Consistently, qRT‐PCR results showed that in the presence of blocked RXRα mRNA expression, vimentin, Snail and ZEB‐1 were up‐regulated but E‐cadherin mRNA expression was down‐regulated (Figure 5D). The results suggested that the lack of RXRα would promote EMT in SW480 cells.

### 3.8 20(S)‐PPD reduced migration and invasion capability in SW620 cells

**Figure 6:**
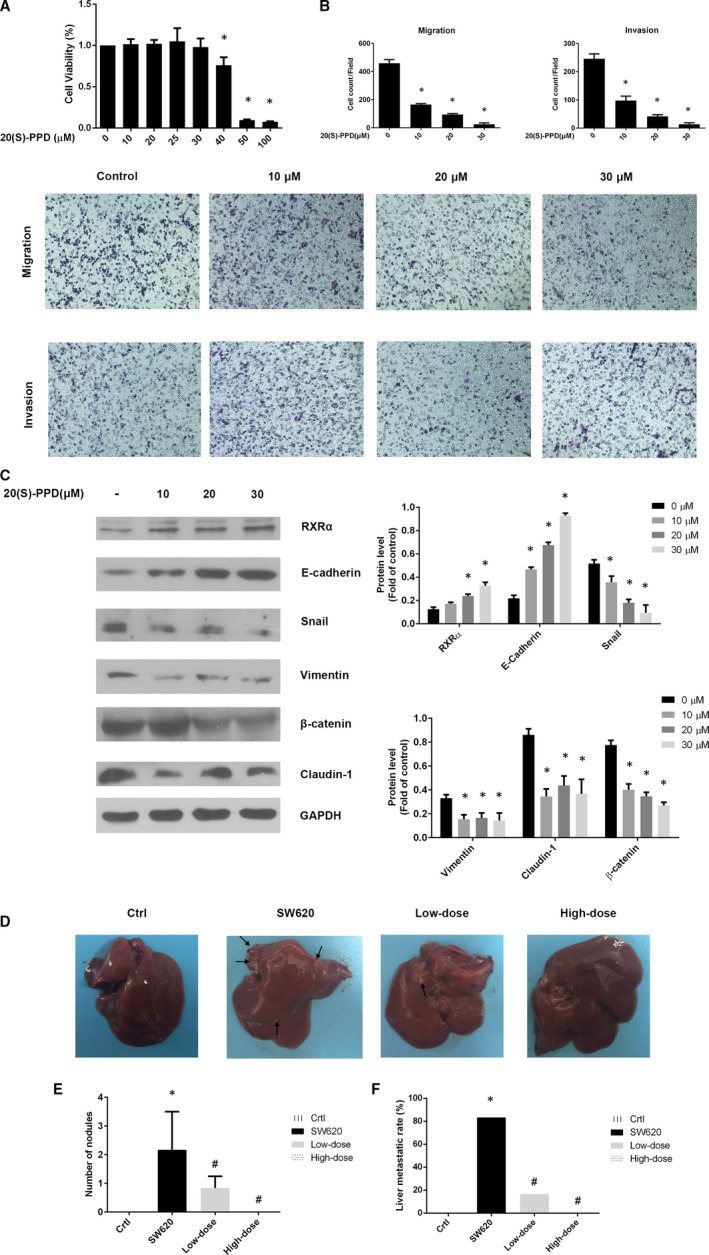
Reduction of epithelial‐mesenchymal transition in SW620 cells by 20(S)‐PPD. A, Viability of SW620 cells after treatment with different concentrations (10, 20, 25, 30, 40, 50 and 100 μmol/L) of 20(S)‐PPD for 24 h (B). The capability of migration and invasion of cells treated with 0, 10, 20 and 30 μmol/L of 20(S)‐PPD (C) Expression of RXRα, E‐cadherin, Snail, vimentin, β‐catenin and Claudin‐1 in SW620 cells with 10, 20 and 30 μmol/L or no 20(S)‐PPD. D, Images of liver metastatic nodules (arrows indicate metastatic nodules) (E) Number of nodules and (F) liver metastatic rate in nude mice. The data represented as mean ± SD. All results were statistically analysed using a two‐tailed Student *t* test. *P < .05 represented a significant difference compared with the control group. #*P *< .05 represented a significant difference compared with the SW620 group. Ctrl, control

In our previous study, we determined the concentration at which 20(*S*)‐PPD showed an anti‐tumour effect,^7^, ^19^, ^20^ but the concentration which shows anti‐metastasis with minimum cytotoxicity in CRC cells needs to be explored. In the MTT assay, SW620 cells were treated with 20(*S*)‐PPD in a dose‐dependent manner, and we found that a significant cytotoxicity exists in 40 μmol/L dosage (Figure 6A). For minimizing the cytotoxic effect, 10, 20 and 30 μmol/L of 20(*S*)‐PPD were selected. The capabilities of migration and invasion in SW620 cells were examined by transwell assay after treating the cells with 10, 20 and 30 μmol/L of 20(*S*)‐PPD. With an increase in the 20(*S*)‐PPD concentration, the migration and invasion capability of the cells decreased in a dose‐dependent manner (Figure 6B). This result suggested that 20(*S*)‐PPD reduced the capability of migration and invasion in SW620 cells in a dose‐dependent manner.

### 3.9 Effect of 20(S)‐PPD on RXRα‐ and EMT‐related proteins expression in SW620 cells

We further examined EMT‐related proteins by Western blot. The expression of RXRα increased with increasing concentrations of 20(*S*)‐PPD in a dose‐dependent manner in Figure 6A. As for EMT‐related proteins, 20(*S*)‐PPD increased E‐cadherin but decreased vimentin, Snail, claudin‐1 and β‐catenin (Figure 6C). These results suggested that 20(*S*)‐PPD repressed EMT by down‐regulating mesenchymal markers while up‐regulating epithelial markers and RXRα expression.

### 3.10 20(S)‐PPD inhibited SW620 cell liver metastasis in vivo

We explored the inhibitory effect of 20(*S*)‐PPD in liver metastasis in the nude mice model. Compared with the control group, SW620 injection caused liver metastasis in the SW620 group, and the 20(*S*)‐PPD decreased liver metastasis (Figure 6D). Both the low‐dose and high‐dose 20(*S*)‐PPD groups had significantly lower liver metastasis occurrence rate and significantly less number of metastatic nodules than the SW620 group (Figure 6E,F). Therefore, 20(*S*)‐PPD performed excellent anti‐metastasis effect in vivo.

### 3.11 Deficient RXRα blocked the 20(S)‐PPD anti‐metastasis effect in SW480 cells

**Figure 7:**
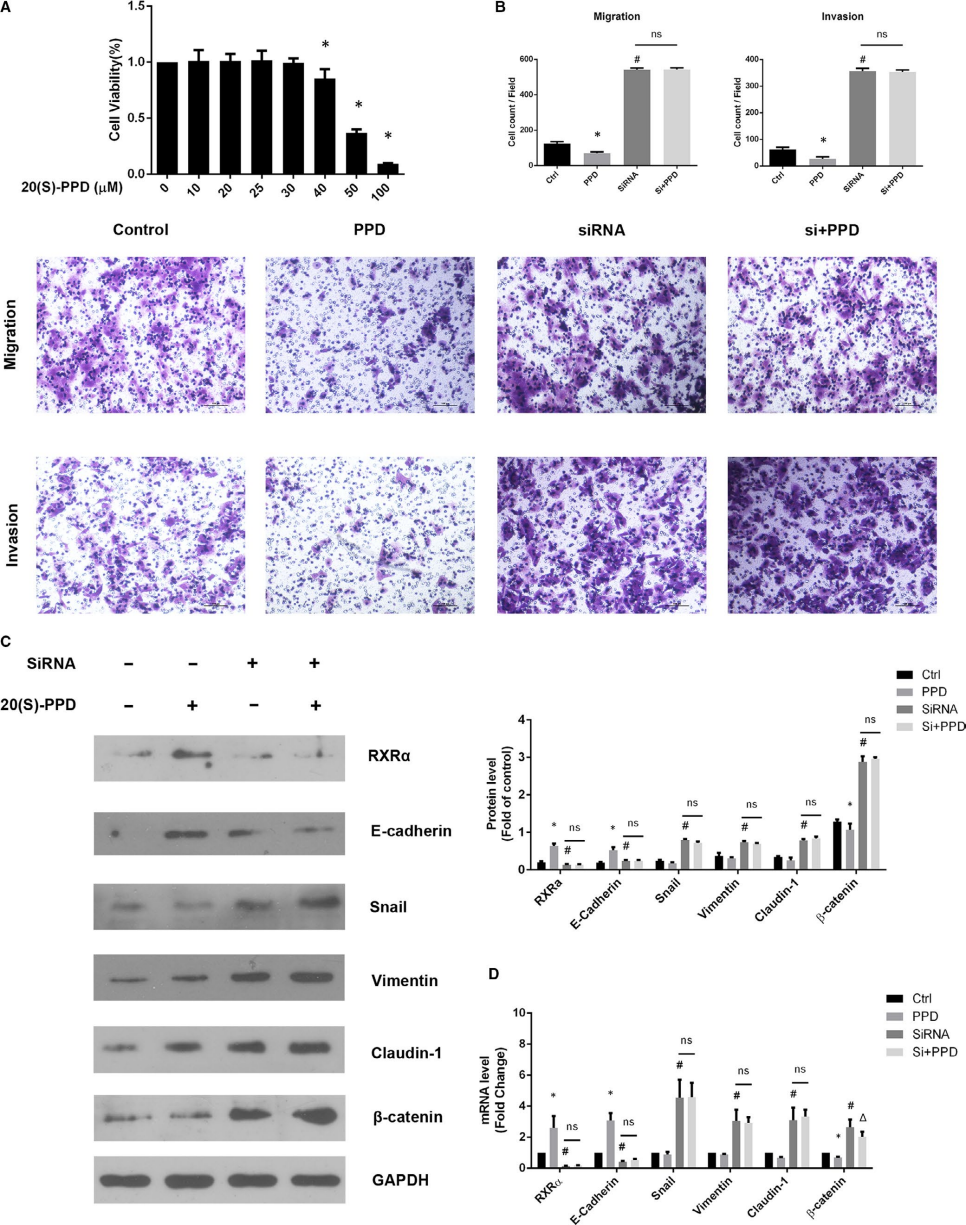
RXRα deficiency blocked the 20(*S*)‐PPD anti‐metastasis effect in SW480 cells. A, SW480 cell viability after treatment with different concentrations (10, 20, 25, 30, 40, 50 and 100 μmol/L) of 20(*S*)‐PPD in 24 h. *P < .05 represented a significant difference compared with the 0 μmol/L group. B, The capability of migration and invasion with treatment with 20(*S*)‐PPD and treated RXRα siRNA. C, Expressions of RXRα, E‐cadherin, Snail, vimentin, β‐catenin and Claudin‐1 and in SW480 cells were analysed by Western blot with 20(*S*)‐PPD and RXRα SiRNA treatment. D, Expression of RXRα, E‐cadherin, vimentin, Snail, claudin‐1 and β‐catenin mRNA in SW480 cell with 20(*S*)‐PPD and RXRα SiRNA treatment. The data represented as mean ± SD. All results were statistically analysed using a two‐tailed Student *t* test. *P < .05 represented a significant difference compared with the control group, #*P *< .05 represented a significant difference compared with the PPD group. Δ*P *< .05 represented a significant difference compared with the SiRNA group. PPD, 20(S)‐PPD; Si, SiRNA; ns, not significant

The results of this study suggest that 20(*S*)‐PPD repressed EMT by regulating RXRα expression. We want to confirm that when RXRα was silenced in SW480 cells, whether 20(*S*)‐PPD would still reduce EMT level. In Figure 5, we proposed that deficient RXRα would promote EMT in SW480 cells. We tested various concentrations of 20(*S*)‐PPD and confirmed that the 20(*S*)‐PPD cytotoxicity effect induction concentration was 40 μmol/L in SW480 cells (Figure 7A). Subsequently, we used 30 μmol/L of 20(S)‐PPD to avoid cytotoxicity in SW480 cells. The results showed that 20(*S*)‐PPD inhibited the migration and invasion capabilities of SW480 cells. Moreover, 20(*S*)‐PPD increased the expression of RXRα and E‐cadherin while decreasing the expression of β‐catenin. However, 20(*S*)‐PPD had no significant effect on the expression of Snail, vimentin and claudin‐1. Interestingly, 20(*S*)‐PPD did not reverse or even reduce the migration and invasion capabilities and affect the expression of EMT‐related proteins after RXRα silencing in SW480 cells (Figure 7B,C). And in mRNA level, 20(*S*)‐PPD could promote RXRα, E‐cadherin while reducing β‐catenin expression. But 20(*S*)‐PPD still could not inhibit EMT level by regulating mRNA level except β‐catenin expression (Figure 7D). The results strongly suggest that 20(*S*)‐PPD repressed the EMT phenotype by regulating RXRα in CRC cells.

## 4 DISCUSSION

As a major transcription factor, NRs regulate almost all biological processes. The disorders in the expression of NRs or the genes those NRs regulated by are important factors in the pathological process of several diseases such as diabetes, obesity, reproductive system diseases, inflammation, cardiovascular diseases and tumours.^21^, ^22^, ^23^ RXRs belong to the NR superfamily and have 3 isoforms: α, β and γ. RXR interacts with several other NRs and plays a central role in the hormonal gene network that regulates many pathways.^24^ The abnormal expression of RXRα was reported in a variety of cancers such as bladder cancer, renal cancer and oesophageal cancer.^25^, ^26^, ^27^ Furthermore, RXRα promotes the proliferation and inhibits the apoptosis of pancreatic cancer cells through TGF‐β/Smad pathway.^28^ It also reported that RXRα was bound by berberine to suppressed β‐catenin in colon cancer.^29^Using retinoids to improve RXRα expression can enhance the sensitivity of prostate cancer to radiotherapy,^30^ although the other two isoforms of RXR, β and γ, also have some anti‐tumour effects.^31^, ^32^, ^33^, ^34^, ^35^, ^36^ However, studies have shown that deficiencies in the α subtype have been shown to have a more detrimental impact on health compared with the other isoforms.^37^, ^38^, ^39^ Abnormal expression and regulation of RXRα may affect a wider range of tissues, organs or tumour types. So the role of β and γ isoforms is much smaller than that of α isoforms. Previous studies reported that there was a lack of RXRα expression in cancer tissues. Consistent with these studies, we found that the expression of RXRα in tumour samples of colorectal cancer patients was lower than that in normal para‐cancer tissues. With respect to clinicopathological sub‐features, there was no significant difference in the expression of RXRα between these two groups at the T1‐T2 stage. Considering the tumour formation time and volume at these stages, we speculated that the expression of RXRα was not been significantly affected. In addition, we showed that RXRα expression was related to lymph node metastasis. According to the American Joint Committee on Cancer, regional lymph node metastasis of CRC patients is at least stage III.^40^ Therefore, lymph node metastasis is an important feature in advanced CRC patients. As EMT is one of the key methods of tumour metastasis, we focused on detecting the differences in the expression of the EMT‐related proteins in cancer and para‐carcinoma tissues. As shown in Figure 1C, cancer tissues had lower E‐cadherin and higher vimentin, Snail and ZEB‐1 expression than para‐carcinoma tissues suggesting that EMT, along with a down‐regulation of RXRα expression, was promoted in cancer tissues. Therefore, the lack of RXRα might be a risk factor for lymph node metastasis.

For tumour cells with metastatic characteristics, the degree of malignancy of tumour cells is determined by their migration and invasion capabilities. The wound healing rate of the scratch and the number of transwell membrane penetrated cells were observed to evaluate the migration capability.^41^ As shown in Figure 2, SW620 cells had lower RXRα expression than SW480 cells. Consequently, SW620 was more malignant than SW480. Consistent with previous studies,^42^, ^43^, ^44^ SW620 cells showed stronger migration and invasion capability than SW480 cells. This supported the association between RXRα expression and the capacity of migration and invasion.

EMT is a critical process in tumour invasion and metastasis, and the degree of EMT is determined by the expression level of epithelial and mesenchymal markers. A study suggested that the highly conserved zinc‐finger transcription factors including Snail, Slug and ZEB‐1 can activate EMT. Moreover, Snail inhibits the expression of E‐cadherin transcription promoter by binding to the E‐box sequence.^45^ These functions are associated with morphological and phenotypic differentiation which promote tumour metastasis. The SW480 cells showed higher E‐cadherin expression but lower N‐cadherin and vimentin expression than the SW620 cells. These results suggested that SW480 cells had a lower degree of EMT than the SW620 cells. More than 80% of colorectal cancers have adenomatous polyposis coli (APC) mutations in the early adenomatous polyp stage.^46^, ^47^ APC accelerated β‐catenin phosphorylation and degradation by ubiquitination in combination with Axin and GSK3β. On the other hand, β‐catenin accumulates in colorectal cancer with mutated APC gene, binds to the transcription factor TCF/Lef and induces tumorigenesis, invasion and metastasis.^48^, ^49^ It was reported that RXRα agonists enhance the interaction between RXRα and β‐catenin and induce APC‐independent β‐catenin degradation.^50^, ^51^ Another study showed that promoting β‐catenin activation up‐regulated claudin‐1 and enhanced colitis‐associated cancer.^52^ Claudin‐1 is a member of the claudin family which belongs to tight junction proteins. In normal epithelium, tight junction proteins maintain cell polarity,^53^ but may have abnormal or high expression in some cancers. Overexpressed claudin‐1 in cancer cells is usually associated with increased invasion and metastatic capabilities ^54^, ^55^ and up‐regulation of ZEB‐1.^56^ Our results showed that the expression of RXRα decreased β‐catenin expression. It was suggested that RXRα might affect EMT by interacting with β‐catenin. The detailed mechanism remains to be further studied. Furthermore, the higher expression of Snail, Slug and ZEB‐1 in SW620 cells than in SW480 cells revealed that SW620 cells had stronger EMT but lower RXRα expression than SW480 cells. Finally, in vivo study showed that SW620 cells were more malignant than SW480 cells. We speculated that reduction in RXRα expression may induce severe EMT.

It was showed that reduced RXRα tended to a higher risk of distant failure after radiotherapy in prostate cancer. However, treatment with the RXRα agonist 9‐*cis*‐retinoic acid restored radiosensitivity.^30^ During EMT process, tumour cells acquired the ability to differentiate into mesenchymal cell and increased drug and chemoradiotherapy resistance.^57^, ^58^ To further validate whether the expression of RXRα affects EMT, RXRα was overexpressed and silenced in SW620 cells and SW480 cells, respectively. The capacity of migration and invasion in RXRα overexpressed SW620 cells were inhibited and EMT‐related factors were decreased. These results suggested that increasing RXRα expression inhibited EMT. In contrast, EMT was reversed in SW480 cells with RXRα siRNA. The migration and invasion capabilities were significantly enhanced, besides the expression of EMT‐related factors at both protein and mRNA levels after silencing RXRα expression. Therefore, the results suggested that RXRα inhibited the process of EMT.

According to recent studies and results from our laboratory, the anti‐tumour effect of 20(*S*)‐PPD was mainly induced by apoptosis through inhibiting Akt phosphorylation, including reducing the expression of phosphorylated GSK3β, which promoted β‐catenin accumulation and intestinal tumorigenesis.^19^, ^59^ We assumed that RXRα may be targeted by 20(*S*)‐PPD, as RXRα can be inhibited by modulating the PI3K/Akt signalling pathway through Akt and FAK phosphorylation in cancer cells.^60^ This study showed that 20(*S*)‐PPD inhibited EMT as well as migration and invasion capabilities and reduced the expression of EMT‐related proteins in SW620 cells in a dose‐dependent manner (Figure 6). In vivo study further demonstrated the inhibitory effect of 20(*S*)‐PPD on liver metastasis induced by SW620 cells. Finally, silencing RXRα disabled the 20(*S*)‐PPD EMT inhibitory effect, and moreover, the cells were unable to restore the expression of EMT‐related proteins and mRNA to normal levels. Interestingly, 20(*S*)‐PPD could still inhibit β‐catenin mRNA expression in Figure 7D. We speculated that 20(*S*)‐PPD down‐regulated β‐catenin mRNA expression in blocking RXRα may be achieved by inhibiting GSK3β phosphorylation. But 20(*S*)‐PPD was unable to degrade accumulated β‐catenin protein.

Our studies have shown that 20(*S*)‐PPD is a multi‐target drug.^7^, ^19^, ^20^, ^61^, ^62^ As for whether the up‐regulated effect of 20(*S*)‐PPD on RXRα is direct needs further study to prove. For example, the expression of upstream and downstream proteins of RXRα were interfered under 20(*S*)‐PPD treatment and then examined RXRα protein and mRNA expression. In conclusion, our study showed that increased RXRα expression was associated with reduced migration, invasion and degree of EMT in metastatic colorectal carcinoma and decreased RXRα expression was associated with high lymph node metastasis. We speculate that RXRα may be a novel indicator of tumour metastasis or a target for inhibiting tumour metastasis by EMT induction. Furthermore, we demonstrated that 20(*S*)‐PPD suppressed EMT and had an anti‐metastasis effect in SW480 and SW620 cells. In addition, these results indicated that 20(*S*)‐PPD inhibited EMT by positively regulating RXRα. In summary, we found that the expression of RXRα had a moderate negative correlation with lymph metastasis and inhibited EMT process. Moreover, 20(*S*)‐PPD can inhibit CRC cells EMT process by regulating RXRα.

## CONFLICT OF INTEREST

The authors declare no conflict of interest.

## AUTHOR CONTRIBUTIONS

**Zeyuan Lu:** Conceptualization (equal); data curation (lead); formal analysis (lead); investigation (equal); software (lead); visualization (equal); writing‐original draft (lead). **Hongyan Liu:** Funding acquisition (equal). **Wenwen Fu:** Data curation (supporting); formal analysis (supporting); investigation (supporting). **Yuchen Wang:** Data curation (supporting); formal analysis (supporting); investigation (supporting). **Jianan Geng:** data curation (supporting); formal analysis (supporting); investigation (supporting). **Yaozhen Wang:** Data curation (supporting); formal analysis (supporting); investigation (supporting). **Xiaofeng Yu:** Investigation (equal); methodology (equal); resources (equal); validation (equal). **Quan Wang:** Conceptualization (equal); methodology (equal); resources (equal); validation (equal); visualization (equal); writing‐review and editing (equal). **Hua‐li Xu:** Conceptualization (equal); funding acquisition (equal); methodology (equal); resources (equal); validation (equal); visualization (equal); writing‐review and editing (equal). **Dayun Sui:** Conceptualization (lead); funding acquisition (equal); methodology (equal); project administration (lead); resources (equal); supervision (lead); validation (equal); visualization (equal); writing‐review and editing (equal).

## Supporting information

Fig S1

## Data Availability

The data sets used and/or analysed during the current study are available from the corresponding author on reasonable request.
