# Supplementary figures and images for: 20(S)‐Protopanaxadiol inhibits epithelial‐mesenchymal transition by promoting retinoid X receptor alpha in human colorectal carcinoma cells
Source: J Cell Mol Med. 2020 Oct 30;24(24):14349–65. doi: 10.1111/jcmm.16054 (PMC7754066; doi:10.1111/jcmm.16054)

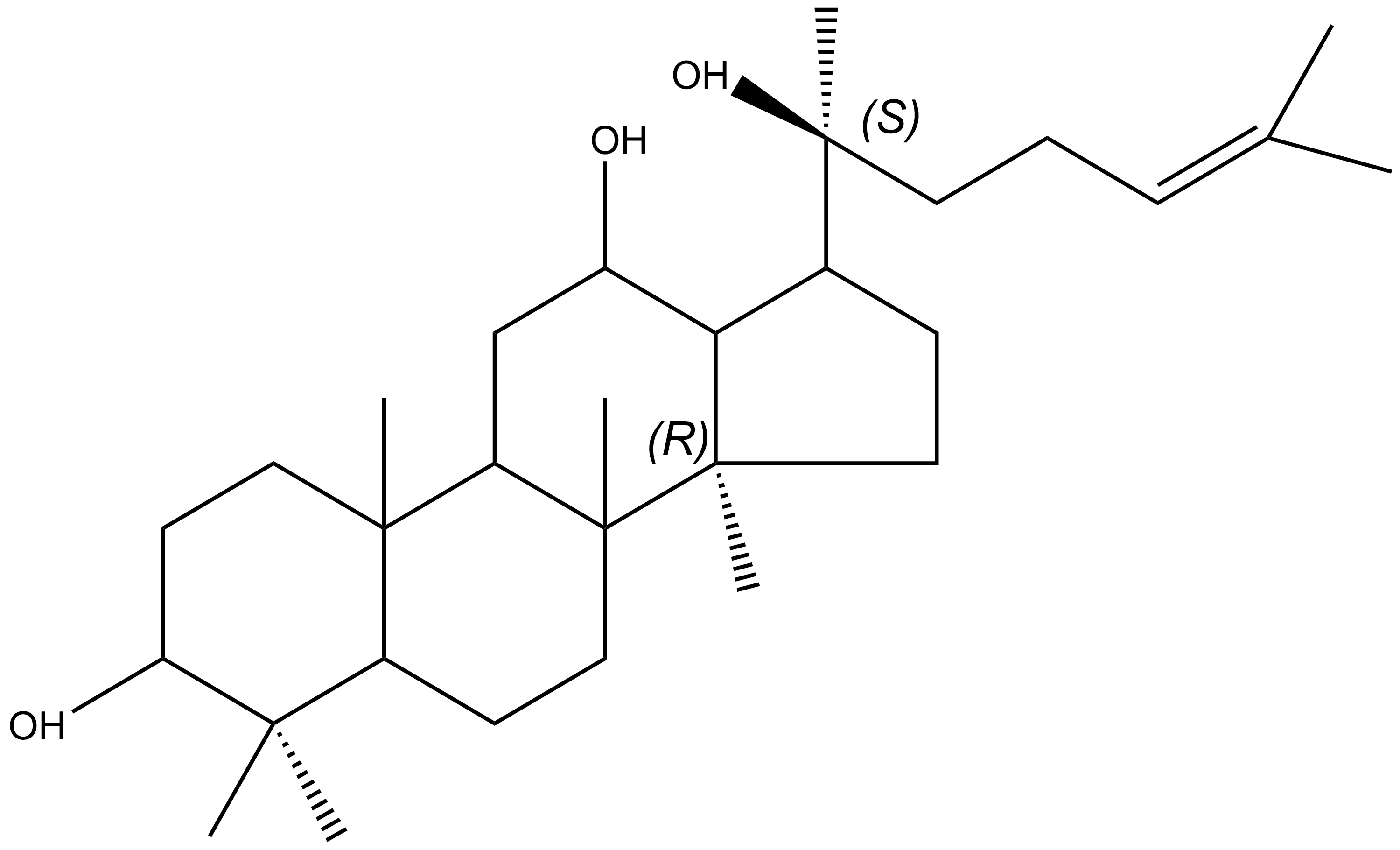

Supplement: Supplementary file 1 — Fig S1 [file JCMM-24-14349-s001.tif]
